# P-2099. A Novel Anaerobic Activity Index to define the “anti-anaerobic” spectrum of antimicrobials

**DOI:** 10.1093/ofid/ofae631.2255

**Published:** 2025-01-29

**Authors:** Natalie A Mackow, David M Aronoff, Vincent B Young, Luther A Bartelt, David van Duin

**Affiliations:** University of North Carolina School of Medicine, Chapel Hill, North Carolina; Indiana University, Indianapolis, IN; University of Michigan, Ann Arbor, MI; University of North Carolina School of Medicine, Chapel Hill, North Carolina; University of North Carolina at Chapel Hill, Chapel Hill, NC

## Abstract

**Background:**

While necessary for the treatment of anaerobic infections, use of antibiotics with anaerobic activity may be associated with poor patient outcomes including graft-versus host disease after bone marrow transplant and mortality in critically ill patients. However, we lack a uniform method to assess the relative anaerobic activity of antibiotics and have poor definitions of what it means for antimicrobials to be “anti-anaerobic”.

**Figure:**
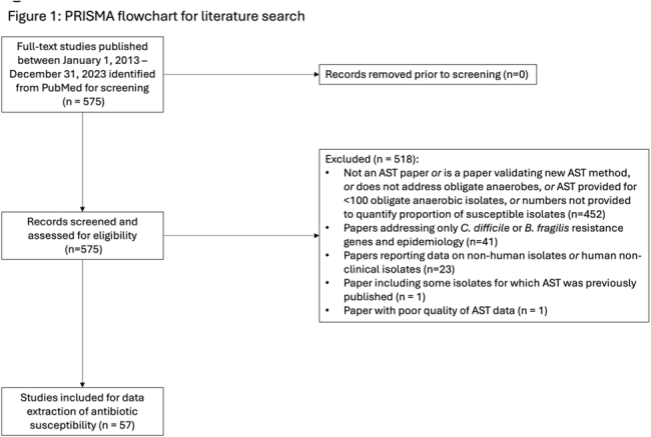

**Methods:**

A PubMed search using terms “microbial sensitivity test”, “anti-bacterial agents” and “bacteria, anaerobic” yielded 575 papers published between January 2013-2024 (Figure 1). Papers with < 100 isolates, non-human isolates, or without antimicrobial susceptibility testing (AST) were excluded. AST for an average of 17,709 anaerobic clinical isolates per antimicrobial from 56 publications were summarized. Anaerobes were categorized into 11 groups based on the literature (Table 1). For 16 commonly used antibiotics, values of 0, 1, or 2 were awarded for each group of anaerobes for average published susceptibility of ≤ 10%, 11-89.4%, and ≥ 89.5%, respectively, and tallied to create a composite score (0-22, Score A). Three alternative scores were created based on different susceptibility cutoffs (Scores B-D; Table 2).

**Table 1:**
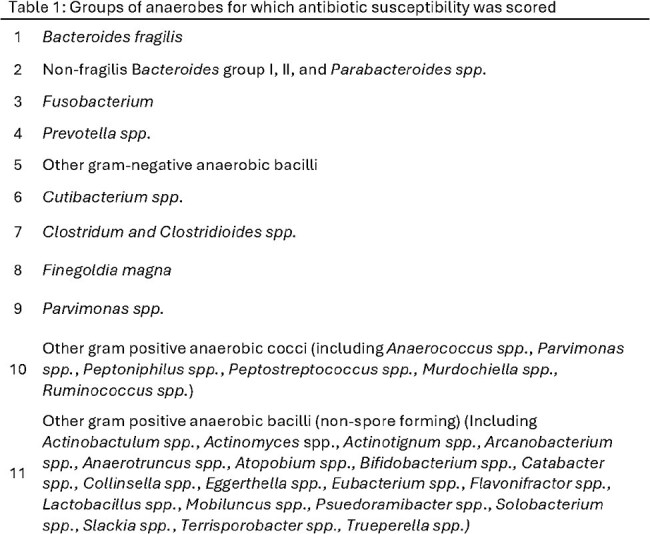
Groups of anaerobes for which antimicrobial susceptibility was scored

**Results:**

In Anaerobic Activity Index Score A, scores ranged from 11 for moxifloxacin to 22 for tigecycline, meropenem, and imipenem (Table 2). Anaerobic Activity Index scores were plotted against the Antibiotic Spectrum Coverage (ASC), a previously published antibiotic “broad” spectrum score (Figure 2). Varying susceptibility thresholds affected estimated anaerobic activity for some antibiotics, including clindamycin and vancomycin, but overall, there was good agreement across all scores (A-D). Moxifloxacin had the lowest overall scores due to high rates of resistance across all groups of anaerobes.

**Figure:**
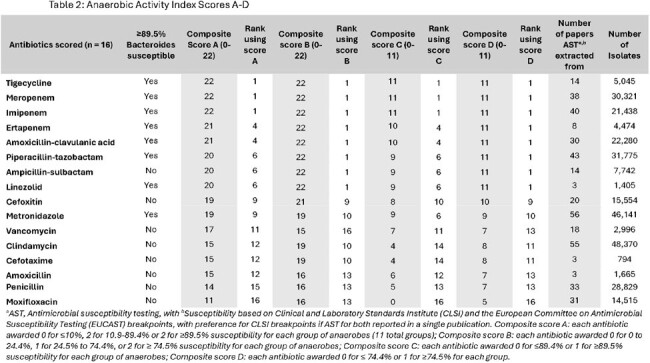

**Conclusion:**

Antibiotic broad-spectrum scores have been proposed, but no valid score exists and no score assessing anaerobic activity has been developed. An Anaerobic Activity Index may improve the definition of “anti-anaerobic” antimicrobials for clinical care and research. Further studies are needed to validate this index as a potential tool to evaluate the effect of anaerobic antibiotic exposure on clinical outcomes.

**Figure:**
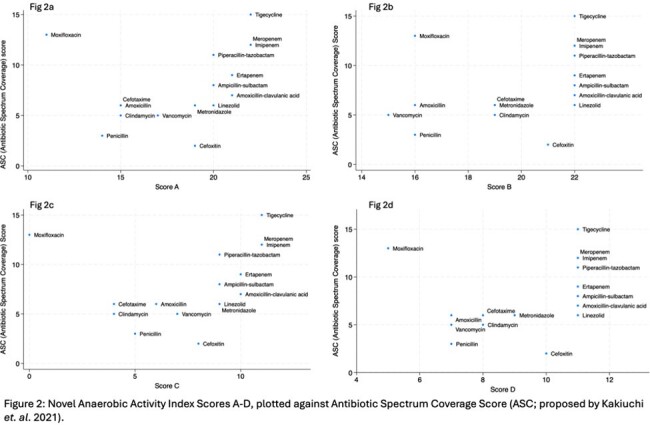

**Disclosures:**

Vincent B. Young, MD, PhD, Aimmune: Honoraria|American Society for Microbiology: Board Member|Debiopharm: Advisor/Consultant|Peggy Lillis Foundation: Board Member|University of Oklahoma COBRE: Advisor/Consultant|Vedanta: Advisor/Consultant|Vedanta Bioscience: Advisor/Consultant|Vedanta Bioscience: Grant/Research Support David van Duin, MD, PhD, Merck: Advisor/Consultant|Merck: Grant/Research Support|Pfizer: Advisor/Consultant|Qpex: Advisor/Consultant|Roche: Advisor/Consultant|Shionogi: Advisor/Consultant|Shionogi: Grant/Research Support

